# Chemistry

**Published:** 1867-10

**Authors:** 


Chemistry. By William Thomas Brande, D.C.L., F.R.S.L., and E. of Her Majesty's Mint; Member of the Senate of the University of London; and Honorary Professor of Chemistry in the Royal Institution of Great Britain. And, Alfred Swaine Taylor, M.D., F.R.S., Fellow of the Royal College of Physicians of London, and Prof. of Chemistry and Med. Jurisprudence in Guy's Hospital. Second American

Edition, thoroughly revised. Philadelphia: HENRY C. LEA. 1867.

This, as its title imports, is a new edition of the large and valuable work on Chemistry, by Drs. Brande and Taylor. It contains 754 closely printed, full sized, octavo pages; and embraces both inorganic and organic chemistry. It is a work of real merit, such as every student and practitioner will find useful, both for study and reference.

For sale by W. B. KEEN & Co., 148 Lake St., Chicago.

